# 15 YEARS OF PARAGANGLIOMA: Clinical manifestations of paraganglioma syndromes types 1–5

**DOI:** 10.1530/ERC-15-0268

**Published:** 2015-08

**Authors:** Diana E Benn, Bruce G Robinson, Roderick J Clifton-Bligh

**Affiliations:** Cancer Genetics, Kolling Institute, Royal North Shore Hospital, University of Sydney, St Leonards, New South Wales, 2065, Australia

**Keywords:** paraganglioma, phaeochromocytoma, renal cancer, gastrointestinal stromal tumour, succinate dehydrogenase

## Abstract

The paraganglioma (PGL) syndromes types 1–5 are autosomal dominant disorders characterized by familial predisposition to PGLs, phaeochromocytomas (PCs), renal cell cancers, gastrointestinal stromal tumours and, rarely, pituitary adenomas. Each syndrome is associated with mutation in a gene encoding a particular subunit (or assembly factor) of succinate dehydrogenase (*SDHx*). The clinical manifestations of these syndromes are protean: patients may present with features of catecholamine excess (including the classic triad of headache, sweating and palpitations), or with symptoms from local tumour mass, or increasingly as an incidental finding on imaging performed for some other purpose. As genetic testing for these syndromes becomes more widespread, presymptomatic diagnosis is also possible, although penetrance of disease in these syndromes is highly variable and tumour development does not clearly follow a predetermined pattern. PGL1 syndrome (*SDHD*) and PGL2 syndrome (*SDHAF2*) are notable for high frequency of multifocal tumour development and for parent-of-origin inheritance: disease is almost only ever manifest in subjects inheriting the defective allele from their father. PGL4 syndrome (*SDHB*) is notable for an increased risk of malignant PGL or PC. PGL3 syndrome (*SDHC*) and PGL5 syndrome (*SDHA*) are less common and appear to be associated with lower penetrance of tumour development. Although these syndromes are all associated with SDH deficiency, few genotype–phenotype relationships have yet been established, and indeed it is remarkable that such divergent phenotypes can arise from disruption of a common molecular pathway. This article reviews the clinical presentations of these syndromes, including their component tumours and underlying genetic basis.

## Introduction

Phaeochromocytomas (PCs) are tumours in the adrenal medulla, and paragangliomas (PGLs) arise in extra-adrenal sympathetic chromaffin tissue or head and neck parasympathetic tissues. Familial occurrence of PGLs was first reported in 1933 by Chase (1933), and co-occurrence of PCs and PGLs (collectively termed PPGLs) in some families was recognised somewhat later (Pritchett 1982, Jensen *et al*. 1991). The genetic basis for hereditary PGL syndrome type 1 (PGL1) was discovered by Baysal *et al*. (2000) by combining knowledge that hypoxia increases the risk of carotid body PGLs with the presence of a hypoxia-responsive gene encoding succinate dehydrogenase subunit D (*SDHD*) within a region at chromosome 11q23 linked in family studies to hereditary head and neck PGLs (HNPGLs). The phenotype of germline *SDHD* mutations was quickly extended to include thoracoabdominal PGLs (TAPGLs) and PCs (Gimm *et al*. 2000, Astuti *et al*. 2001*a*). Soon thereafter, the *SDHC* gene was found to be mutated in familial HNPGLs (PGL3; Niemann & Muller 2000) and *SDHB* mutations were discovered in familial PPGLs (PGL4; Astuti *et al*. 2001*b*). *SDHAF2*, required for flavination of SDHA, is mutated in the rare PGL2 (Hao *et al*. 2009), and PGL5 is associated with mutations in *SDHA* (Burnichon *et al*. 2010).

Germline mutations in predisposition genes are now found in 25–30% of PPGLs overall (Gimenez-Roqueplo *et al*. 2012). Germline mutations in *SDHx* genes are the commonest genetic cause of PPGLs, occurring in up to 25% cases (Baysal *et al*. 2002, Neumann *et al*. 2002). By comparison the next most commonly associated genes are von Hippel–Lindau (*VHL*) (4–10%), *RET* (1–5%) and *NF1* (1–5%).

SDH is located on the inner mitochondrial membrane and functions in the mitochondrial respiratory chain and the Krebs cycle. In the respiratory chain, SDH transports electrons to the ubiquinone pool, then to cytochrome *c* of complex III. In the Krebs cycle, SDH catalyses conversion of succinate to fumarate. Therefore, two predictable consequences of SDH inactivation are succinate accumulation and increased production of reactive oxygen species. Both outcomes have been suggested to contribute to cellular accumulation of hypoxia-inducible factors (Selak *et al*. 2005), and tumours associated with SDH deficiency display notable upregulation of hypoxia-responsive genes. Notably, this gene expression signature is shared by PPGLs associated with mutations in *VHL* (Dahia *et al*. 2005).

**Figure 1:**
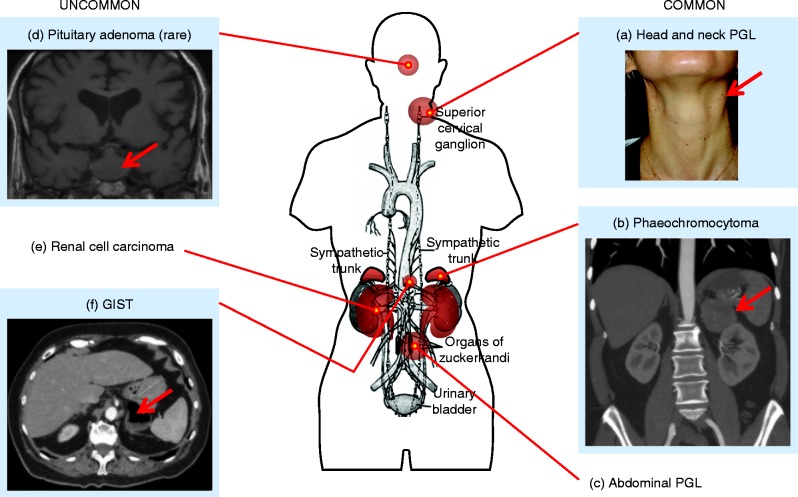
Tumour types associated with paraganglioma syndromes 1–5. In each panel the tumour is arrowed and is: (A) a left-sided carotid body paraganglioma (the scar from previous surgery for a right carotid body PGL is faintly visible); (B) a left-sided phaeochromocytoma; (C) abdominal PGL (not shown); (D) a pituitary macroadenoma; (E) renal cell carcinoma (not shown), and (F) a gastrointestinal stromal tumour.

In this article, we review the clinical manifestations of PGL1–5. We begin by considering the approach to the patient and then discuss each syndrome in turn, including genotype–phenotype interrelationships, before a detailed overview of each component tumour type. We finish with a proposed clinical management approach. A summary of clinical features of each syndrome is shown in Table 1, and a synoptic view of PGL-associated tumours is shown in Fig. 1.

## Cardinal manifestations of PGL1–5

PGL1–5 are characterised by the development of PGLs and/or PCs, together with a variable risk of developing renal cancers, gastrointestinal stromal tumours (GISTs) or (rarely) pituitary tumours. The patient with a PGL syndrome will therefore present in one of four ways: i) with symptoms and/or signs of catecholamine excess; ii) with symptoms and/or signs of local tumour mass; iii) as an incidental finding on an imaging study for unrelated purpose and iv) after genetic testing in context of familial disease.

Clinical features of catecholamine excess include hypertension, headache, sweating, palpitations, and often morbid apprehension or anxiety. These symptoms often come in paroxysms that can last for minutes or hours, with variable frequency. Clinical examination may reveal hypertension (although absent in 10–20% cases, and paroxysmal in 30%), pallor, hyperhidrosis and tremor (Plouin *et al*. 1981).

Rarely, patients may present with catecholaminergic ‘crisis’ accompanied by acute cardiomyopathy and severe hypertension (but sometimes with shock), and/or multiorgan failure, lactic acidosis, encephalopathy, fever and hyperglycaemia (Amar & Eisenhofer 2015). In such cases, precipitating factors may be present including recent use of dopamine D2 agonists (e.g. metoclopramide), corticosteroids, β-blockers or anaesthesia.

PGL of the urinary bladder is associated with catcholaminergic symptoms that are provoked by micturition, and may also be associated with painless haematuria (Beilan *et al*. 2013).

Clinical features of local tumour mass are relevant for HNPGLs and also for metastatic PPGLs. Carotid body tumours and vagal PGLs may present with neck fullness, cough, hoarse voice, or dysphagia and are clinically palpable in the lateral upper neck (Fig. 1). Glomus tympanicum tumours may present with unilateral hearing loss and/or pulsatile tinnitus. Jugular PGLs may present with palsies of lower cranial nerves VII, IX, X, XI and/or XII (Suárez *et al*. 2013). Local symptoms from tumour mass also occur with renal cell cancers that can present with flank pain and/or haematuria, and GISTs that may present with abdominal pain and/or gastrointestinal bleeding. Pituitary tumours are rarely associated with hereditary PGLs and present with features either of local tumour mass (headache, visual field deficit) and/or hormonal excess (acromegaly, hyperprolactinaemia; Xekouki *et al*. 2015).

Incidental discovery of PPGLs on imaging is becoming an increasingly important mode of their diagnosis; for instance, the proportion of PCs detected incidentally is now 25–30% of all cases (Mannelli *et al*. 1999, Amar *et al*. 2005, Kopetschke *et al*. 2009, Shen *et al*. 2010). Specific imaging findings are discussed in more detail below.

A detailed family history is essential, including known history of PPGLs, renal cancer, GISTs and/or unexplained sudden death.

## The PGL syndromes 1–5

Some general points can be made about these syndromes: i) they are autosomal dominant disorders, with maternal imprinting effects for *SDHD* and *SDHAF2*; ii) the penetrance of tumour development in subjects carrying *SDHx* mutations is highly variable, in particular for PGL3–5 – highlighted by the occasional finding that a child or young adult is the index case in a family where the parent (and sometimes grandparent) carrying the pathogenic allele has not developed tumours and iii) tumour development does not clearly follow a predetermined pattern – PPGLs, renal cancer, GISTs and/or pituitary tumours occur in a seemingly random fashion in affected subjects.

### PGL1: SDHD

*SDHD* is maternally imprinted, with the result that the disease almost only ever occurs with paternally inherited mutations (Taschner *et al*. 2001, Neumann & Erlic 2008, Pigny *et al*. 2008, Yeap *et al*. 2011) and as such, to the uninitiated, may appear to ‘skip’ generations. When paternally inherited, *SDHD* mutations are associated with frequent development of HNPGL and less commonly TAPGLs or phaechromocytomas (Neumann *et al*. 2004, Schiavi *et al*. 2005, Benn *et al*. 2006, Ricketts *et al*. 2010). PGL1 has very high lifetime penetrance, and 75% of carriers will manifest disease by age 40 (Benn *et al*. 2006). PGL1 demonstrates multifocal tumour development in around 56% cases, although malignancy is infrequent (Neumann *et al*. 2004, Schiavi *et al*. 2005, Benn *et al*. 2006, Ricketts *et al*. 2010). Renal cancers are found in 8% (Ricketts *et al*. 2010). Pituitary adenomas have been reported in a few cases of PGL1 (Xekouki *et al*. 2012, Evenepoel *et al*. 2014).

**Figure 2:**
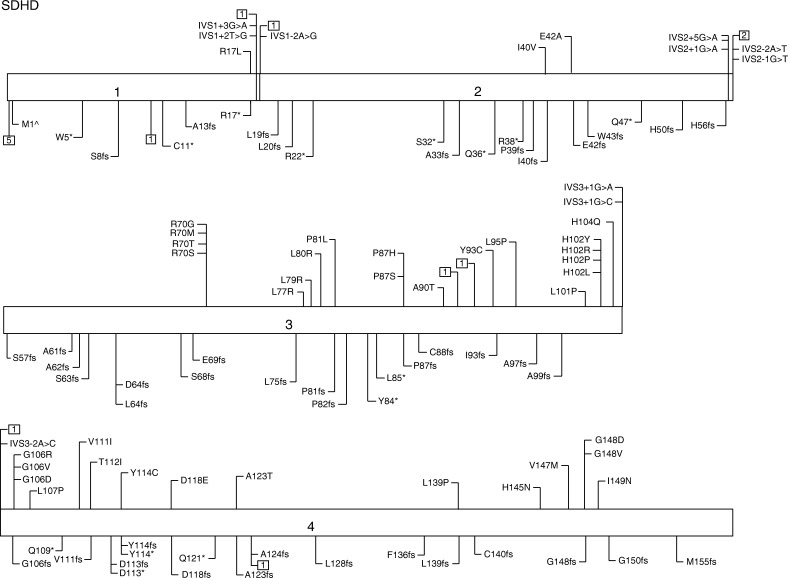
PGL1 due to mutations in *SDHD*. Genotypes associated with paraganglioma syndromes. Mutations are not represented by standard nomenclature; abbreviations are used for representation purposes only. Not all mutations identified worldwide have been included. M1^∧^, mutation in initiator methionine indicating loss of transcript; *, represent stop codons; □□, represent a large deletion/insertion/duplication and the number indicates the number of different mutations at that site. Data from http://chromium.lovd.nl/lovd_sdh, Bayley *et al*. (2005), McWhinney *et al*. (2004).

More than 130 unique DNA mutations have been reported in PGL1 (Fig. 2). These mutations are evenly distributed across the four coding exons. Prematurely truncating mutations (by frameshift or nonsense variants) are particularly common in *SDHD*, and one study found that these mutations were associated with a significantly increased risk of phaeochromocytoma or sympathetic PGL compared to missense mutations that were not predicted to impair protein stability (Ricketts *et al*. 2010).

### PGL2: SDHAF2

PGL2 was initially described in a Dutch family (van Baars *et al*. 1982), and associated with *SDHAF2* in 2009 (Hao *et al*. 2009). *SDHAF2* mutations are a rare cause of PPGLs: only four PGL2 families have been described. *SDHAF2* is encoded by four exons located at chromosome 11q13 and, like *SDHD*, is maternally imprinted. HNPGLs occur in 75% carriers of paternally inherited mutations, starting from relatively young age (earliest affected aged 22 years) and often multifocal but are not malignant (Kunst *et al*. 2011). Neither TAPGLs nor PCs have yet been reported in PGL2.

### PGL3: SDHC

Mutations in *SDHC* have been identified in patients with HNPGL and, rarely, TAPGLs and PCs (Schiavi *et al*. 2005). Linkage analyses of large families revealed this third locus for hereditary PGL, termed PGL3 (Niemann & Muller 2000, Niemann *et al*. 2003, Muller *et al*. 2005). Overall, germline *SDHC* mutations are found in around 4% of HNPGL (Schiavi *et al*. 2005) but very few functioning PPGLs (Mannelli *et al*. 2007, Peczkowska *et al*. 2008). Patients with *SDHC* mutations are more likely to develop carotid body tumours, less likely to have multiple tumours than in *SDHD* mutated PGL, and have low malignant potential compared to *SDHB*-mutated PGL (Schiavi *et al*. 2005).

**Figure 3:**
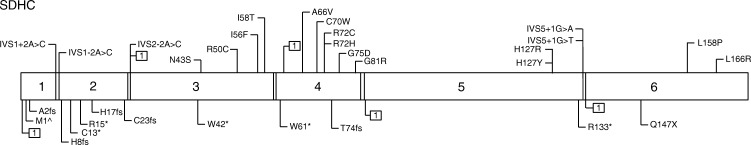
PGL3 due to *SDHC* mutations. Genotypes associated with paraganglioma syndromes. Mutations are not represented by standard nomenclature; abbreviations are used for representation purposes only. Not all mutations identified worldwide have been included. M1^∧^, mutation in initiator methionine indicating loss of transcript; *, represent stop codons; □□, represent a large deletion/insertion/duplication and the number indicates the number of different mutations at that site. Data from http://chromium.lovd.nl/lovd_sdh, Bayley *et al*. (2005).

Nearly 50 unique *SDHC* mutations have been described in PGL3 to date, and these are evenly distributed between the six coding exons (Fig. 3).

### PGL4: SDHB

*SDHB* mutations were first found to be associated with PPGL in 2001 (Astuti *et al*. 2001*b*, Gimenez-Roqueplo *et al*. 2001). In comparison to PGL1, patients with *SDHB* mutations have lower penetrance for disease and may present with unifocal disease at a later age, with perhaps only about 40% of carriers manifesting the disease by age 40 (Schiavi *et al*. 2010). However, PGL4 is more likely to be associated with TAPGL and/or PC, is more likely to present with symptoms from local tumour mass, and malignant disease occurs in around a third of patients (Benn *et al*. 2003, Gimenez-Roqueplo *et al*. 2003, Brouwers *et al.* 2006, Amar *et al*. 2007, Timmers *et al*. 2007). HNPGL occurs in 20–30%, renal cell cancer in about 14% and GISTs in 2% of carriers of pathogenic *SDHB* mutations (Benn *et al*. 2006, Ricketts *et al*. 2010).

**Figure 4:**
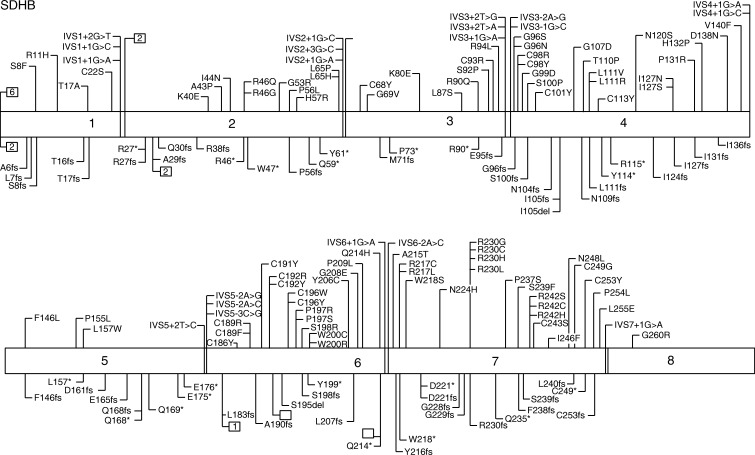
PGL4 due to *SDHB* mutations. Genotypes associated with paraganglioma syndromes. Mutations are not represented by standard nomenclature; abbreviations are used for representation purposes only. Not all mutations identified worldwide have been included. M1^∧^, mutation in initiator methionine indicating loss of transcript; *, represent stop codons; □□, represent a large deletion/insertion/duplication and the number indicates the number of different mutations at that site. Data from http://chromium.lovd.nl/lovd_sdh, McWhinney *et al*. (2004), Bayley *et al*. (2005), Cascón *et al*. (2006).

More than 200 unique *SDHB* mutations occurring in all its eight coding exons have been described in PGL4; there are interesting clusters of mutations at the junction of exons 3/4 and in exons 6 and 7 that appear to occur within iron–sulfur cluster domains of SDHB (Fig. 4).

### PGL5: SDHA

Mutations in *SDHA* were originally described as a cause of autosomal recessive juvenile encephalopathy (Leigh syndrome; Bourgeron *et al*. 1995). In 2010, a heterogygous *SDHA* germline mutation was identified in a 32-year-old woman with abdominal PGL (Burnichon *et al*. 2010). *SDHA* mutations remain a rare cause of PPGL, accounting for about 3% of cases and with low penetrance such that familial disease is uncommon (Korpershoek *et al*. 2011). GISTs and pituitary adenomas may also be the presenting features of PGL5 (Dwight *et al*. 2013*a*,*b*).

**Figure 5:**
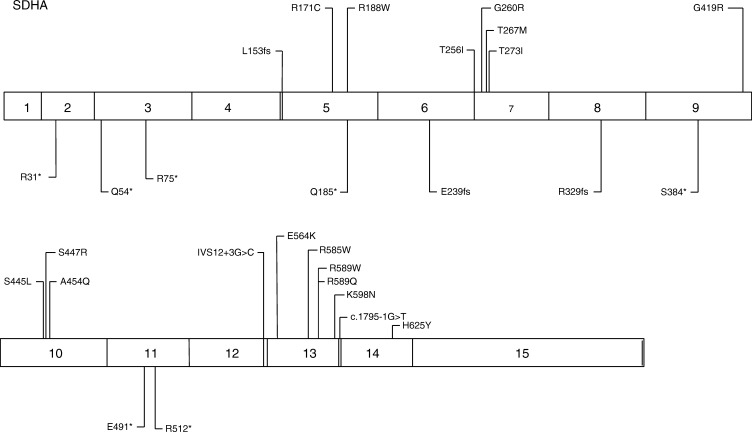
PGL5 due to *SDHA* mutations. Genotypes associated with paraganglioma syndromes. Mutations are not represented by standard nomenclature; abbreviations are used for representation purposes only. Not all mutations identified worldwide have been included. M1^∧^, mutation in initiator methionine indicating loss of transcript; *, represent stop codons; □□, represent a large deletion/insertion/duplication and the number indicates the number of different mutations at that site. Data from http://chromium.lovd.nl/lovd_sdh, Bayley *et al*. (2005).

Genetic testing for *SDHA* mutations is complicated by the presence of three pseudogenes – *SDHAP1* (localized to 3q29), *SDHAP2* (3q29) and *SDHAP3* (5p15.33) – which are highly homologous to not only the coding regions of *SDHA* but also the intronic regions of the gene. *SDHA* is also the largest *SDHx* gene, with 15 exons (Fig. 5). Fortunately, SDHA immunohistochemistry has proved useful in identifying tumours that are likely to contain *SDHA* mutations (Burnichon *et al*. 2010).

## Tumours associated with PGL1–5

### Paragangliomas

These tumours arise from the neuroendocrine paraganglia that occur along the paravertebral axis from the base of the skull through to the pelvis (Fig. 1), and are divided into those that derive from the parasympathetic paraganglia (HNPGLs) or those from sympathetic paraganglia (TAPGLs). Approximately 40% of all PGLs are associated with SDH deficiency (Gill *et al*. 2010*a*), and those associated with *SDHB* mutations (PGL4) are at higher risk of malignancy (Timmers *et al*. 2007).

HNPGLs associated with PGL syndromes include carotid body PGLs located at the carotid bifurcation, glomus vagale tumours located along the vagus nerve and glomus jugulare tumours located in the jugular foramen (Gimenez-Roqueplo *et al*. 2013, Taïeb *et al*. 2014). Rarer locations for HNPGLs include glomus tympanicum tumours within the middle ear and PGLs of the larynx, nasopharynx, orbit, tongue and thyroid (Taïeb *et al*. 2014). HNPGLs are usually non-secreting: only about 5% are associated with elevated plasma or urine normetanephrine, although 30% are associated with elevated dopamine metabolite 3-methoxytyramine (van Duinen *et al*. 2010, Eisenhofer *et al*. 2012). HNPGLs are also less likely to be malignant (<5%; Taïeb *et al*. 2014). Living at high altitude may promote the development of HNPGLs in PGL1 (Astrom *et al*. 2003); whether altitude is a phenotypic modifier in other PGL syndromes has not been established. HNPGLs may be discovered as an incidental finding on imaging studies. Magnetic resonance imaging (MRI) is typically the best modality for detailed imaging of HNPGLs, demonstrating low T1-signal and intermediate-high signal on T2-weighted images and intense enhancement after gadolinium contrast; on computed tomography (CT) imaging, intratumoral blood vessels are strongly enhanced after contrast (Taïeb *et al*. 2014).

**Figure 6:**
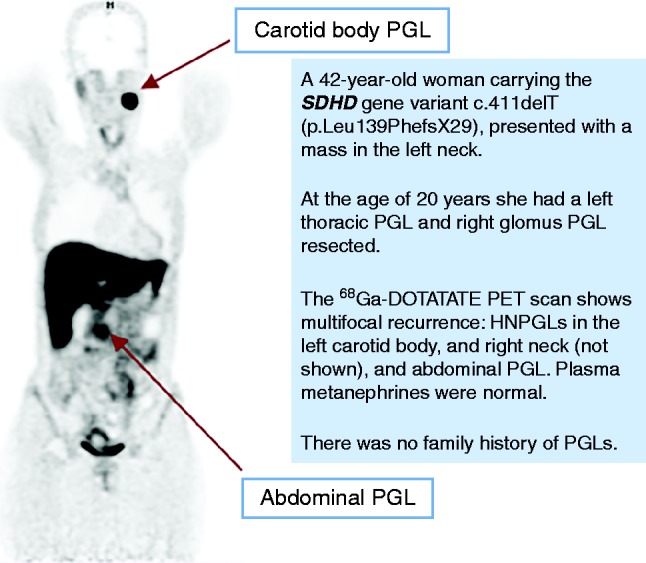
A representative clinical vignette of PGL syndrome type 1.

TAPGLs arise from chromaffin tissue in sympathetic ganglia in the abdomen, less commonly in the pelvis (including urinary bladder) and rarely in the mediastinum (Young 2006). Within the abdomen, a common location is the organ of Zuckerkandl at the origin of the inferior mesenteric artery. TAPGLs display broadly similar imaging characteristics to PCs on CT and MRI. More recently, ^68^Ga-DOTATATE positron emission tomography (PET)/CT has shown great promise in imaging both primary and metastatic TAPGLs (Maurice *et al*. 2012). In a series of 17 subjects with SDHB-related metastatic PGLs, DOTATATE PET identified 98.6% metastatic lesions compared to 85.8% for FDG–PET (Janssen *et al*. 2015). A PGL1 case history illustrating the utility of ^68^Ga-DOTATATE scanning is shown in Fig. 6.

### Phaeochromocytomas

These are catecholamine-secreting PGLs confined to the adrenal medulla. Approximately 3% of all PCs are associated with SDH deficiency (Gill *et al*. 2010*b*) and occur as part of PGL1 and PGL4; except for negative SDHB immunohistochemistry (discussed in detail below), these are otherwise histologically indistinguishable from sporadic PCs or from PCs associated with other heritable syndromes (e.g. multiple endocrine neoplasia type 2 (MEN2), VHL and NF). PCs that occur in PGL1 and PGL4 secrete only noradrenaline (and/or dopamine), in contrast to PCs associated with MEN2, NF1 or MAX from which mixed adrenaline and noradrenaline secretion occurs. PCs associated with *SDHB* mutations (PGL4) show a higher risk of malignancy.

PCs are usually easily visible (≥3 cm) on abdominal CT at time of presentation and are typically dense (≥10 Hounsfield units), but may be heterogeneous, with areas of low density. On MRI, PCs are classically isointense with respect to the liver on T1-weighted images and hyperintense on T2-weighted images (Jacques *et al*. 2008).

Nuclear medicine imaging with MIBG or PET may also be useful for diagnosis of PC in adrenal lesions with equivocal biochemical testing. MIBG is less sensitive in smaller adrenal lesions such that tumors <2.5 cm are likely to be negative (Bhatia *et al*. 2008). Standard ^18^F-FDG–PET imaging was reported to have 88% sensitivity in diagnosis of non-metastatic PC/PGL (Timmers *et al*. 2009), although this series included a relatively large number of tumors containing *SDHB* mutations (which are more likely to be positive due to altered glucose transport). Other PET tracers such as ^18^F-DOPA (Hoegerle *et al*. 2002) and ^18^F-FDA (Pacak *et al*. 2001) have shown utility in PPGL diagnosis and these may be more useful than MIBG in assessment of smaller adrenal lesions. As discussed above for HNPGLs and TAPGLs, ^68^Ga-DOTATATE PET imaging may also be useful for diagnosis of PC (although the normal adrenal medulla does take up this tracer) and/or metastatic disease.

### Renal cell cancer

Renal carcinoma occurs in ∼14% of PGL4 (Vanharanta *et al*. 2004, Ricketts *et al.* 2008, Gill *et al.* 2011) and 8% of PGL1; one case with *SDHC* mutation has been reported, and none with *SDHA* or *SDHAF2* (Gill *et al*. 2013). It may be the index tumour in PGL1 or PGL4, presenting at mean age of 37 years (range 14–76), and indeed in many cases it is the sole tumour: two-thirds of patients with SDH-deficient renal cancers had not developed metachronous PPGLs or GISTs by time of report, although many of these patients were still at relatively young age (Gill *et al*. 2014*a*, Paik *et al.* 2014). Bilateral renal cancers occur in 26% of SDH-deficient cases, and metastases occur in 33% of the cases. Although only 0.05–0.2% of all renal cancers are associated with *SDHx* mutations, they can be recognised by distinct pathological features including solid or focally cystic growth, uniform cytology with eosinophilic flocculent cytoplasm, intracytoplasmic vacuolations and inclusions and round-to-oval low-grade nuclei. In *SDHB* mutated renal cancers, immunohistochemistry for SDHB is negative. Although no clear-cut genotype–phenotype correlations have been defined, it is interesting to note that four unrelated subjects who developed renal cancer all harboured the same *SDHB* splice site mutation (c.423+1G>A), and that two of these subjects developed multifocal disease (Gill *et al*. 2014*a*). Further work is required to determine whether this mutation is specifically associated with risk of developing renal cancer.

### Gastrointestinal stromal tumours

GISTs are the commonest mesenchymal tumor of the gastrointestinal tract, and most are driven by somatic activating mutations of KIT (75–80%) or PDGFRA (5–8%) (Corless *et al*. 2011). GISTs associated with hereditary PGL syndromes are usually detected by negative immunohistochemistry for SDHB (see below). However, only 50% of such SDH-deficient GISTs are found to be associated with germline mutations in an *SDHx* gene: 30% due to *SDHA* mutations, and 10–20% due to mutations in *SDHB*, *SDHC* or *SDHD* (Gill *et al*. 2010*a*). SDH-deficient GISTs occur exclusively in the stomach, commonly metastasise to lymph nodes, have a propensity to multifocal or metachronous disease, commonly show primary resistance to imatinib and demonstrate a tendency to relatively indolent behavior of metastases (Gill *et al*. 2010*a*). GISTs associated with hereditary PGL due to germline mutations of *SDHA*, *SDHB*, *SDHC* or *SDHD* are known as the Carney–Stratakis syndrome (Pasini *et al.* 2008, Janeway *et al*. 2011, Pantaleo *et al*. 2011*a*,*b*). GISTs also occur as part of the Carney triad: the syndromic but non-hereditary association of SDH-deficient GISTs now known to be associated with hypermethylation of the *SDHC* promoter (Haller *et al*. 2014, Killian *et al*. 2014).

### Pituitary tumours

An aetiopathological link between *SDHx* mutations and pituitary tumours is strongly suggested by case reports of pituitary tumours that demonstrate loss of SDHB immunostaining, occurring in patients who carry germline mutations in *SDHA* (Dwight *et al*. 2013*b*, Papathomas *et al*. 2013), *SDHB* (Xekouki *et al*. 2015), *SDHC* (Lopez-Jimenez *et al*. 2008) and *SDHD* (Xekouki *et al*. 2012). Nevertheless, pituitary adenomas are very uncommon in PGL syndromes (Xekouki & Stratakis 2012), and conversely <0.3% of all pituitary tumours are associated with SDH deficiency (Gill *et al*. 2014*b*). Based on the small number of reports available, SDHx mutation-associated pituitary tumours are more commonly macroadenomas secreting either growth hormone or prolactin, and display a more aggressive phenotype (Xekouki *et al*. 2015).

### Thyroid carcinoma

A few individuals with an *SDHB* or an *SDHD* pathogenic variant have had thyroid carcinoma (Neumann *et al*. 2004, Ricketts *et al*. 2010). The association is generally regarded to be casual between a common disease (thyroid cancer) and a rare one (PPGL).

### Negative immunohistochemistry for SDHB is a surrogate marker for SDH deficiency and provides functional validation of pathogenic *SDHx* mutations

*SDHx* are tumour suppressor genes: inheritance of a pathogenic mutation on one allele in the germline is typically accompanied by loss of the normal allele in tumours (Gimenez-Roqueplo *et al*. 2001). Loss of SDHB immunostaining has proved to be an important tool for recognising tumours associated with mutations in any of the *SDHx* genes, and indeed is a robust assay in all the multiple tumour types described above. In a large multicenter study, 62 of 69 PPGLs associated with mutations in *SDHB*/*C*/*D*/*AF2* were negative for SDHB immunohistochemistry, whereas two *SDHD*-mutated tumours were scored as immunopositive (Papathomas *et al*. 2015). About 16% of *VHL*-mutated PPGLs also show loss of SDHB staining (Papathomas *et al*. 2015). Tumors associated with mutations in *RET* or *NF1*, on the other hand, usually show positive granular SDHB cytoplasmic staining (consistent with normal mitochondrial location of SDH; van Nederveen *et al*. 2009, Gill *et al*. 2010*b*). Immunohistochemistry for SDHA has also been used to identify tumors associated with germline mutations in that gene (Burnichon *et al*. 2010, Korpershoek *et al*. 2011, Papathomas *et al*. 2015).

Extended experience suggests that <10% of PC/PGLs are SDHB negative by immunohistochemistry but are not associated with identifiable *SDHx* mutations (Papathomas *et al*. 2015), raising the possibility that other mechanisms of mitochondrial complex 2 instability exist which lead to tumorigenesis. As noted above, this phenomenon is more common in GISTs.

## Genetic counseling/testing

Despite high heritability, the approach to genetic testing in patients presenting with PPGLs remains controversial, acknowledged in recent guidelines that recommend the use of a clinical feature-driven diagnostic algorithm to guide specific genetic testing (Lenders *et al*. 2014). When a patient presents with an apparently solitary PPGL, the opportunity to diagnose an underlying hereditary basis is supported by positive implications for that patient (early detection of metachronous disease and/or associated tumours) and their family members, but counterbalanced by variable penetrance and the need for lifelong screening. This is particularly true for *SDHA* (PGL5) and *SDHC* (PGL3), for which penetrance appears to be much lower than for *SDHB* (PGL4) and *SDHD* (PGL1). In contrast, PCs are rarely the index event in other forms of hereditary PC because their syndromic features are more highly penetrant: MEN2 (associated with *RET* mutations) will almost always present with medullary thyroid cancer, Von Recklinhausen's disease (*NF1*) will be apparent from cutaneous stigmata of that disease and a diagnosis of VHL syndrome is known in about 50–70% cases before PPGLs develop (Opocher *et al*. 2005).

The approach to genetic testing is therefore moderated by several factors, including age at presentation, location of tumour, malignant disease, presence of syndromic features and/or multifocal disease, pattern of circulating catecholamines and immunohistochemistry of tumour.

Consistent with autosomal dominant pattern of inheritance, each child of an individual with a hereditary PGL syndrome has a 50% chance of inheriting the pathogenic variant. Parent-of-origin effects on disease expression for *SDHD* and *SDHAF2* have been described above, such that children inheriting these mutations from their mother have negligible risk of developing the disease.

Preimplantation genetic diagnosis is an option that some *SDHx* carriers will wish to consider, although careful counseling about its merits is always required.

## Clinical management

The following general principles are advocated in managing patients with hereditary PPGL syndromes (Lenders *et al*. 2014):

- diagnosis is based on clinical suspicion, followed by confirmatory biochemistry (elevated plasma normetanephrine in the case of TAPGL or PC) and imaging (CT or MRI);
- if hereditary PGL is known or strongly suspected in a patient presenting with an index tumour, then imaging from neck to pelvis should be performed to exclude synchronous lesions; ^68^Ga-DOTATATE PET imaging may be appropriate in this regard;
- definitive treatments should be planned and performed at expert centres;
- for catecholamine-secreting tumours (TAPGLs, PCs and some HNPGLs) surgery is appropriate but only after pre-operative treatment with an α-blocker (e.g. phenoxybenzamine or doxazosin);
- treatment options for non-secreting HNPGLs include surgery, radiosurgery, radiofrequency ablation or cryoablation (Taïeb *et al*. 2014);
- histopathology should include careful assessment of SDHB and SDHA immunohistochemistry;
- negative SDHB immunohistochemistry should prompt consideration of genetic testing for mutations in *SDHA*, *SDHB*, *SDHC* or *SDHD* after appropriate genetic counseling; negative SDHA immunohistochemistry should prompt consideration of genetic testing for mutations in *SDHA*;
- genetic testing is performed on DNA extracted from peripheral blood leucoytes and should include validated methods for detecting point mutations, insertions and deletions as well as large deletions in *SDHx* genes;
- a positive result from genetic testing should lead to cascade testing of first-degree relatives after appropriate counseling and
- individuals discovered to carry a pathogenic mutation in *SDHx* genes should undergo lifelong biochemical and clinical surveillance for PPGLs. For PGL1 and PGL4, imaging from neck to pelvis (e.g. with MRI) every 2–3 years is recommended to detect PPGLs, renal cell cancers and GISTs.

## Conclusions

Elucidating the genetic basis of the hereditary paraganglioma syndromes has stimulated great advances in clinical care for these patients, providing opportunities for early detection and treatment of component tumours, but not without costs: both in terms of resources required for genetic testing and then lifetime screening of *SDHx* mutation carriers to detect tumour development anywhere from base of skull to pelvis; and also from the psychological burden these patients bear from not knowing if, when, where and in what manner (benign or malignant) these tumours will develop. Further research is needed to clarify if certain genotypes more reliably predict phenotypic behavior, or if environmental and/or genetic modifiers can be incorporated into risk algorithms. There is also an urgent need to develop better therapies for metastatic paragangliomas; and in these, SDH deficiency may yet prove to be an Achilles heel susceptible to synthetically lethal treatments.

## Footnote

This paper is part of a thematic review section on 15th Anniversary of Paraganglioma and Pheochromocytoma. The Guest Editors for this section were Wouter de Herder and Hartmut Neumann.

## Figures and Tables

**Table 1:** Clinical features (penetrance) of PGL syndromes 1–5

**Syndrome**	**Gene**	**PC**	**TAPGL**	**HNPGL**	**Multifocal**	**Malignant**	**RCC**	**Other**
PGL1	*SDHD*^a^	∼10–25%	20–25%	85%	55–60%	∼4%	∼8%	GIST and PA
PGL2	*SDHAF2*^a^	0	0	100%	0	0	0	–
PGL3	*SDHC*	0	Rare	?^b^	15–20%	0%	Rare	GIST
PGL4	*SDHB*	20–25%	50%	20–30%	20–25%	∼30%	∼14%	GIST and PA
PGL5	*SDHA*	Rare	Rare	Rare	Rare	Rare	0	GIST and PA

PC, phaeochromocytoma; TAPGL, thoracoabdominal PGL; HNPGL, head and neck PGL; RCC, renal cell carcinoma; PA, pituitary adenoma; GIST, gastrointestinal stromal tumour. Neumann *et al*. (2002), Amar *et al*. (2005), Schiavi *et al*. (2005), Benn *et al*. (2006), Cascón *et al*. (2009), Hao *et al*. (2009), Mannelli *et al*. (2009), Burnichon *et al*. (2009), Ricketts *et al*. (2010), Welander *et al*. (2011) and Gimenez-Roqueplo *et al*. (2012).

a Paternally inherited.

b Lifetime prevalence not yet determined.
